# Incidental occurrence of neutropenia in children hospitalised for COVID-19

**DOI:** 10.1186/s13052-022-01234-5

**Published:** 2022-03-15

**Authors:** Francesco Folino, Camilla Menis, Giada Maria Di Pietro, Raffaella Pinzani, Paola Marchisio, Samantha Bosis

**Affiliations:** 1 University of Milan, Via Festa del Perdono 7, 20122 Milan, Italy; 2 Fondazione IRCCS Ca’ Granda Ospedale Maggiore Policlinico, Milan, Italy

**Keywords:** COVID-19, Neutropenia, Children

## Abstract

**Background:**

Investigations on haematological alterations in paediatric COVID-19 have been focused mostly on lymphocytes and clotting profiles. Neutropenia has been occasionally reported and its course and impact on the disease have not been elucidated. The aim of this study was to describe the epidemiology, course, and impact of neutropenia in children with COVID-19 hospitalised in a tertiary care referral paediatric ward.

**Methods:**

A single-centre retrospective study was conducted. Hospitalised children between 1 month and 18 years with confirmed COVID-19 and neutropenia were included and compared to non neutropenic patients. Complete blood picture with differential blood count, serum biochemistry, clotting profiles were performed; clinical data, length of hospitalisation, and prescription of drugs were collected.

**Results:**

Twelve out of 95 patients (12.63%) with documented SARS-CoV-2 infection were neutropenic and met the inclusion criteria. The mean age was 161 days (range 38—490 days). The mean duration of symptoms in neutropenic children was 3.82 days, while the mean length of hospitalisation was 7.67 days. These findings were not significantly different in the two study groups. All patients had mild clinical manifestations and were discharged without sequelae.

**Conclusions:**

We provided the first comprehensive study on neutropenia in mild paediatric COVID-19 infection. Our findings show that the main features of this haematological disorder in COVID-19 are analogous to the well-known transient benign neutropenia associated with other common viral infections. In our setting, neutropenia does not emerge as a potential negative prognostic factor in paediatric COVID-19.

## Introduction

Severe acute respiratory syndrome coronavirus 2 (SARS-CoV-2), the new coronavirus, is responsible for a respiratory disease now defined as coronavirus disease (COVID-19) [1]. The severity of this condition rises with age, as hospitalisation rate and mortality are higher in older patients, while serious disease and death are relatively rare in children and young adults [2, 3]. Children and younger patients infected with SARS-CoV-2 are indeed frequently asymptomatic or experience milder symptoms including fever, cough, pharyngitis, gastrointestinal symptoms, and changes in sense of smell or taste [4, 5].

In the adult population, lymphopenia has been reported as a frequent biological disorder in patients with COVID-19 and as a predictor factor of the severity of the disease [3]. Concerning the paediatric population, data on laboratory findings associated with COVID-19 remain limited. Among the haematological alterations, neutropenia seems to be one of the altered parameters recorded in paediatric cases, differently from adults [6–11]. Nevertheless, incidental neutropenia is commonly observed in paediatric practice and it is mostly connected to viral infections [12, 13]. Viral-associated neutropenia is related to virus-induced redistribution of neutrophils from the circulating to the marginating pool; it usually occurs after 24–48 h of illness and persists for 3–8 days, which generally corresponds to the period of viremia [12–14]. To date, evidence on transient isolated neutropenia in children with SARS-CoV-2 infection is lacking. Venturini A et al. described two cases of isolated severe transient neutropenia in two infants and pointed out the relevance of a complete blood count monitoring in these patients [13].

The aim of this study was to describe the epidemiology, course, and impact of neutropenia in children hospitalised for COVID-19 in a tertiary care referral paediatric ward.

## Methods

### Study design and subjects

A single-centre retrospective study was conducted in the Paediatric Highly Intensive Care Unit, Fondazione IRCCS Ca' Granda Ospedale Maggiore Policlinico, a tertiary care referral paediatric ward located in Milan, Italy.

Inclusion criteria were the following: 1) Hospitalised infants and children aged 1 month—18 years admitted from the 21st of February 2020 to 20th of February 2021; both sexes were included; 2) Symptomatic SARS-CoV-2 infection confirmed by a positive reverse-transcriptase-polymerase-chain-reaction (RT-PCR) assay of a nasopharyngeal aspirate or swab; 3) Neutropenia detected by routine complete blood count defined as Absolute Neutrophil Count (ANC) less than 1.50 × 10^3^/mm^3^.

Exclusion criteria included: 1) Other primary diagnoses known to cause neutropenia (i.e., cancer/leukaemia, congenital bone marrow failure syndromes, hypersplenism); 2) Children treated with agents known to cause neutropenia as a side effect (i.e. anti-epileptic treatment).

Moreover, employing the same criteria, hospitalised children with symptomatic SARS-CoV-2 infection and without neutropenia were included in the analysis as a control group.

### Laboratory examination

All patients included in the study were subjected to a complete blood picture with differential blood count. Leukopenia was defined as a total white blood count (WBC) of less than 4.0 × 10^3^/mm^3^. Neutropenia was defined as ANC of less than 1.50 × 10^3^/mm^3^ cells; it was defined as mild with an ANC of 1.00–1.50 × 10^3^/mm^3^, moderate with an ANC of 0.50–1.00 × 10^3^/mm^3^, severe with an ANC < 0.50 × 10^3^/mm^3^ [15]. Serial blood counts were performed for each patient and the minimum, maximum, and mean values were obtained for each parameter. The minimum value of the minimums, the maximum value of the maximums, and the average value between the averages calculated for each patient were therefore extrapolated. Complete serum biochemistry analysis including C-reactive protein (CRP), hepatic and kidney function was performed; prothrombin time (PT), activated partial thromboplastin time (APTT), fibrinogen, and D-dimer levels were measured. All patients included were screened for viral and bacterial infections to exclude any coinfections; in particular, a screening of viral infections by serological tests and swabs and a screening of bacterial agents by cultures of blood, urine, and stool cultures were performed.

The same analyses have been repeated after the onset of neutropenia to exclude any sovrainfection.

### Clinical evaluation

Clinical data were collected, including age, sex, medical history, comorbidities, frequency and severity of infections, and exposure to drugs. A full clinical examination was performed daily. The evidence of COVID-19 was evaluated in terms of time from onset of the first symptom to admission, duration of symptoms and recovery, clinical signs and symptoms, and severity of infection. We also evaluated the length of hospitalisation, the prescription of drugs, the clinical severity during the hospital stay evaluated by using the PEWS score (Paediatric Early Warning Score) [16].

### Data and statistical analysis

Continuous variables are presented as maximum, minimum, means (standard deviations (SD)), as appropriate, whereas discrete variables are described using absolute and relative frequencies.

Independent sample T-test was used for continuous variables and Chi-squared test for categorical variables. All calculations were performed using SPSS software (version 12, Chicago, IL).

## Results

During a period of one year, 839 children were admitted to our paediatric ward. As a standard procedure, a nasopharyngeal swab or aspirate for SARS-CoV-2 was performed in each patient before admission. 95 children resulted positive and of them, 19 presented neutropenia during the infectious episode.

Among these, 7 were excluded: two of them had been transplanted (kidney and liver transplant) and were being treated with immunosuppression schemes; one of them had leukaemia and was being treated with chemotherapy drugs; two of them developed neutropenia after the treatment with piperacillin-tazobactam; one of them developed a COVID-19 associated pneumonia and showed neutropenia after treatment with hydroxychloroquine and lopinavir/ritonavir; one of them was known to have pancytopenia, before admission, undergoing further diagnosis.

**Fig. 1:**
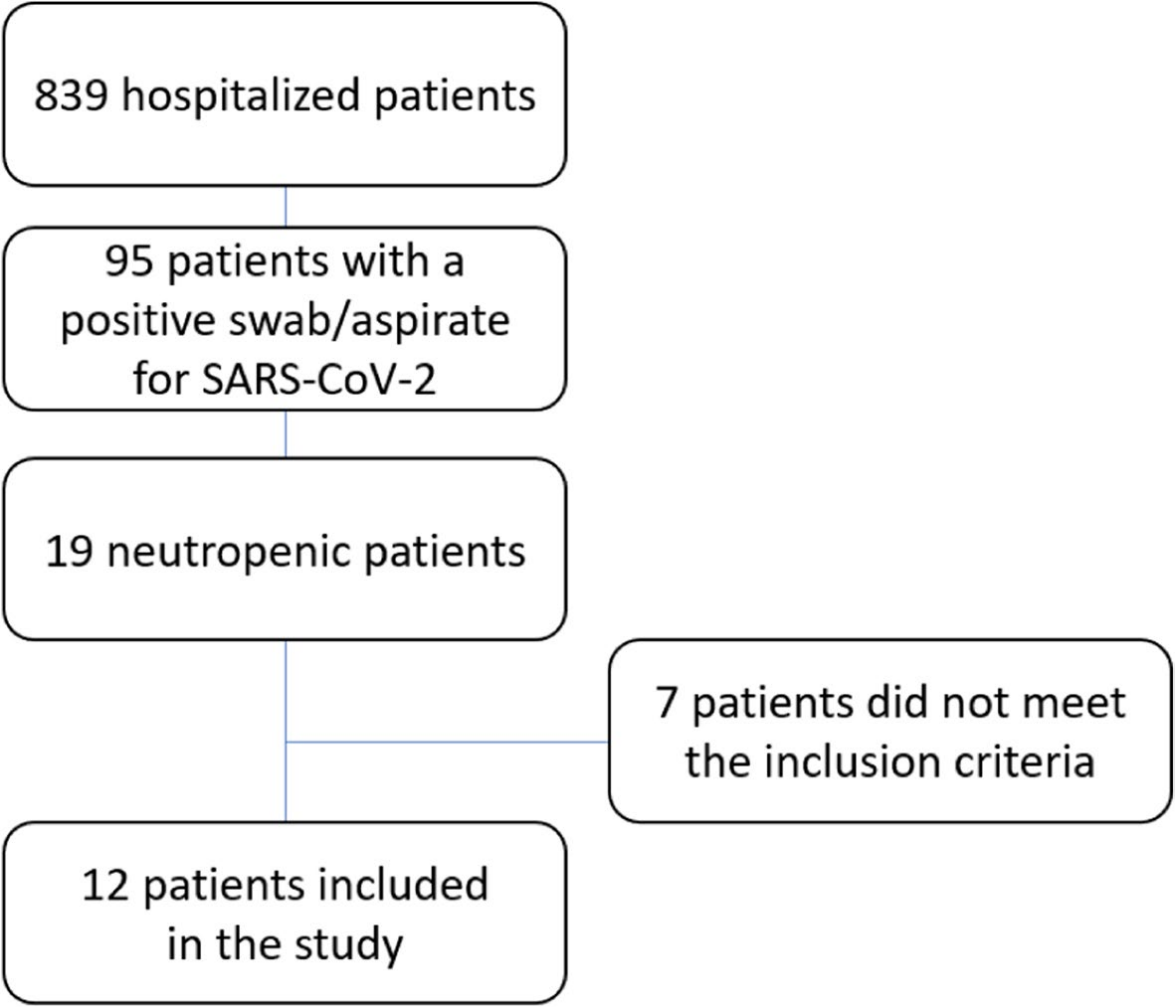
Neutropenic patients included in the study

Therefore, 12 neutropenic patients were included in the study as shown in Fig. 1. Hence, the total frequency of neutropenia among the hospitalised children with COVID-19 in our setting was estimated to be 12.63%. As a control group, 12 non-neutropenic children with similar age, hospitalised for symptomatic SARS-CoV-2 infection, were included in the analysis.

The included 12 neutropenic patients were 11 males and 1 female, compared to the control group which comprehended 5 males and 7 females. In the neutropenic group, age ranged from 38 to 490 days (mean and SD 160.58 ± 136.51 days), while non neutropenic children were slightly older, as their age ranged from 33 to 1127 days (mean and SD 328.67 ± 353.69 days), without statistically significant difference between the two groups (*p* = 0.147).

Among the neutropenic patients, only 1 out of 12 presented leukopenia, while the others showed a normal white blood count. 3 children presented a mild neutropenia, 5 presented a moderate form, and 4 a severe form. The minimum ANC value was 0.16 × 10^3^/mm^3^ (Table 1).

**Table 1:** Complete blood picture with a differential blood count of the neutropenic patients included. The minimum value of the minimums, the maximum value of the maximums, and the average value between the averages calculated from the serial blood counts performed for each patient were extrapolated; *p* values were calculated considering mean values

	**Neutropenic**			**Non-neutropenic**			***p***
	**Minimum**	**Maximum**	**Mean (SD)**	**Minimum**	**Maximum**	**Mean (SD)**	
**Leukocytes (× 10**^**3**^**/mm**^**3**^**)**	2.68	15.47	7.63 (2.34)	4.5	27.52	12.00 (4.45)	0.06
**Neutrophil (× 10**^**3**^**/mm**^**3**^**)**	0.16	7.05	1.45 (0.98)	1.52	15.12	5.60 (2.87)	** < 0.001**
**Lymphocytes (× 10**^**3**^**/mm**^**3**^**)**	1.87	11.53	5.41 (1.57)	1.98	16.84	5.34 (3.00)	0.95
**Monocytes (× 10**^**3**^**/mm**^**3**^**)**	0.16	2.92	0.89 (0.45)	0.24	4.84	1.24 (0.62)	0.12
**Basophil (× 10**^**3**^**/mm**^**3**^**)**	0	0.07	0.03 (0.01)	0.01	0.08	0.37 (0.16)	0.081
**Red Blood Cells (× 10**^**6**^**/mm**^**3**^**)**	3.16	5.00	4.09 (0.31)	2.66	5.39	4.24 (0.77)	0.515
**Haemoglobin (g/dl)**	8.70	14.00	11.08 (1.24)	8.40	13.5	11.36 (1.39)	0.384
**Hematocrit (%)**	24.00	37.00	31.42 (2.71)	24.6	38.1	32.45 (3.89)	0.49
**MCV (fl)**^a^	68.00	85.00	77.83 (5.49)	66.4	92.8	77.43 (8.45)	0.93
**MCH (pg)**^b^	22.60	31.00	27.58 (2.54)	22.8	33.6	27.2 (3.28)	0.73
**MCHC (pg)§**	33.00	38.00	35.67 (1.23)	33.30	36.6	35.1 (0.94)	0.18
**Platelets (× 10**^**3**^**/mm**^**3**^**)**	62.00	642.00	376.25 (69.4)	196.0	1054.0	446.8 (158.9)	0.18
**MPV (fl)°**	8.50	13.00	10.24 (1.01)	8.4	11.4	9.8 (0.62)	0.25

^a^*MCV* Mean corpuscular volume

^b^*MCH* Mean corpuscular haemoglobin, *§ MCHC* Mean corpuscular haemoglobin concentration, *MPV* Mean platelet volume

Concerning the timing of occurrence, neutropenia was found after a period ranging from 0 days (concurrently with clinical onset) to 13 days from the first clinical manifestation (mean and SD 3.38 ± 3.24 days).

Concerning the other cell lines, 3 out of 12 infants from each group were anaemic, without significant differences in mean haemoglobin values (*p* = 0.384).

Two out of 12 (16.6%) of our neutropenic patients presented thrombocytopenia (nadir 62 × 10^3^/mm^3^ and 122,000 × 10^3^/mm^3^); none of the children included in the control group had thrombocytopenia, while thrombocytosis was detected in 4 out of 12 children in this cohort.

In the neutropenic group, CRP values ranged from 0 to 3.08 mg/dl with a mean and SD of 0.68 ± 0.73 mg/dl; higher mean CRP values were detected in the control group (range 0 to 19.25 mg/dL, mean and SD 2.09 mg/dL ± 3.35); however, this difference did not show statistical significance (*p* = 0.198).

The remaining parameters (ferritin, AST, ALT, GGT, Cr, urea, CK, and LDH, clotting profiles, fibrinogen and D-dimer levels) were normal in all of the 24 children included in the study.

Among neutropenic children, the screening for viral and bacterial infections identified 2 children infected with rhinovirus and 2 with rotavirus, while no bacterial infections were identified at admission or during hospitalisation in this cohort; in the control group, 3 rhinovirus infections and 3 urinary tract infection by *E. coli* were detected.

Regarding the medical history of the neutropenic patients included in the study, only one infant showed a positive history for comorbidities: carbamoyl-phosphate synthetase 1 deficiency (CPS1-D), a disorder of urea cycle metabolism characterised by hyperammonemia that occurs a few days after birth [17]. However, this pathology is not associated with neutropenia or haematological disorders in general. Similarly, only one child in the control group had comorbidities, as this patient suffered from bronchodysplasia.

Eleven infants out of the 12 identified neutropenic children presented with febrile illness, compared to 5 out of 12 non-neutropenic children (*p* = 0.03)

Six neutropenic infants also had diarrhoea, 3 had rhinitis, 2 had cough and 1 had pharyngodynia; in the control group, 2 out of 12 also presented with diarrhoea, 1 with rhinitis, 3 with cough. None of these clinical manifestations was significantly different between the two groups.

At the admission and during the hospital stay patients had complete cardiovascular, respiratory, abdominal, and central nervous systems evaluation every day. Regardless of the grade of neutropenia, all patients presented a mild course of the disease, persisted in good clinical conditions, and recovered briefly (PEWS score persistently = 0).

Among non neutropenic children, one patient underwent surgery for intestinal intussusception and had a regular post surgical course; the other children included had a mild course of the disease and recovered briefly, analogously to neutropenic patients. The mean duration of symptoms was 4.41 days, compared to 3.82 days reported in neutropenic patients (*p* = 0.61).

The therapies administered to the patients included in the study are summarised in Table 2.

**Table 2:** Specific therapeutic interventions

Therapy	Neutropenic children	Non-neutropenic children
Paracetamol	6	**5**
Cefotaxime	3	2
Ceftriaxone	0	2
Amoxicillin with Clavulanate	3	3
Metronidazole	0	1
Hydrochlorothiazide / Spironolactone	0	1
5% Glucose and Salts (Nacl)	2	3
Salbutamol Aerosol	2	2
Methylprednisolone	1	1
No Therapy	2	1

The length of hospitalisation in the group of neutropenic children varied from 3 to 30 days (mean and SD 7.67 ± 7.49); the patient suffering from CPS1-D had a longer hospital stay (30 days) as he underwent further diagnostic examination for his condition after the resolution of SARS-CoV-2 infection. The mean length of hospital stay in this group after excluding this patient was 5.65 days.

Similarly, in the control group the length of hospitalisation ranged from 3 to 23 days (mean and SD 8.83 ± 6.55); however, in this group one patient had a longer hospital stay for social issues (23 days); after excluding this child, the mean length was 7.5 days. No significant difference was found comparing the mean hospital stay of the study groups (*p* = 0.904).

Concerning duration of neutropenia, 8 out of 12 patients in the related group were discharged with a mild neutropenia, after documenting an increasing ANC count trend on blood tests and confirming their good clinical condition; unfortunately, follow up data are not available for those patients. 2 more patients were found neutropenic at the first blood test; thus, we cannot define the actual duration of neutropenia without a previous normal examination. In the remaining two patients neutropenia lasted 3 and 6 days. Of note, regardless of the severity of neutropenia, the resort to stimulating therapy was not necessary for our population.

## Discussion

To our knowledge, our study provides the first detailed analysis on the course and clinical impact of neutropenia in mild paediatric COVID-19 on a small cohort of patients.

Neutropenia is a common incidental finding in several viral infections in childhood and has been occasionally reported in SARS-CoV-2 paediatric infection, but its course and impact on the disease have not been fully elucidated. Moreover, several studies on paediatric COVID-19 have considered the total WBC count with a focus on lymphocytes, often not providing data on ANC. At present, existing data show that the preponderance of children with COVID-19 has a normal WBC count and that lymphopenia is rarer in children than in adults [18, 19].

When haematological anomalies were identified in children with COVID-19, leukopenia and lymphopenia were the most recurrent findings [20, 21], while in neonates and infants lymphocytosis was more frequently observed [22]. The overall frequency of neutropenia among children hospitalised for SARS-CoV-2 infection in our study was 12.63%, resulting lower compared to data available in the literature so far, which define rates of 23%—26% [19, 23].

Henry BM et al. provided a comprehensive review on the laboratory alterations in paediatric COVID-19, reporting a pooled prevalence of neutropenia of 38% among 610 patients included in 24 studies [8]. However, these findings are not easily comparable, as some of the studies define neutropenia with different cut-offs (i.e., ANC < 2 × 10^3^/mm^3^), most of the evidence consists in case reports/case series, methods are not always clearly defined and exclusion criteria are often less stringent, thus the prevalence of neutropenia can be overestimated in some cases.

Interestingly, our population of neutropenic children showed a lower mean age compared to patients without neutropenia. The evidence available does not provide insights concerning a possible correlation between age and neutropenia related to COVID-19. Venturini A et al. reported two cases of severe transient neutropenia in two infants aged 23 and 39 days in the course of mild COVID-19 [13], while another report described transient neutropenia in 3 neonates (aged 16–31 days) [24]. In line with these findings, our results show that incidental neutropenia during mild COVID-19 infection often involves younger children under 2 years of age, similarly to most of the common viral infections [12, 14, 25]. Thus, in children, not only the clinical seriousness but also the age may have an impact on the ANC during SARS-CoV-2 infection. Furthermore, the evidence of neutropenia in infants could be an indicator of the age-related diverse immunologic reaction to SARS-CoV-2 infection, as previously highlighted in other reports [13, 26].

Concerning the severity of neutropenia, 3 out of 12 patients (25%) presented a mild form, 5 (41.7%) presented a moderate form, and 4 (33.3%) a severe form. As neutropenia in paediatric COVID-19 has not been investigated, data on its severity in this condition are lacking. An important communication on the potential occurrence of severe neutropenia in paediatric COVID-19 has been provided by an Italian group in a report describing two infants with mild SARS-CoV-2 infection who experienced severe neutropenia (nadir 0.24 × 10^3^/mm^3^ and 0.48 × 10^3^/mm^3^) in absence of other alterations in blood test and experiencing a benign clinical course with spontaneous recovery [13]. Studies on larger cohorts of neutropenic patients hospitalised for various conditions, different from COVID-19, define lower rates of severe neutropenia, ranging from about 9% to 15% [27, 28]; however, data provided by Husain EH et al. on a cohort of 55 patients with infection-related neutropenia describe a distribution more similar to our results (mild neutropenia 6%, moderate neutropenia 51%, severe neutropenia 43%) [14].

Cohort studies and meta-analysis available on paediatric COVID-19 describe a mean hospital stay ranging from 5 to 12.5 days, with a significantly longer duration in children under 5 years old [29, 30]. Specific data on neutropenic patients are lacking. A case series by White A et al. provides an initial insight on this topic, describing 3 neutropenic neonates with SARS-CoV-2 infection who were hospitalised for a short period, ranging from 77 to 81 h [24]. In our study, the mean duration of hospitalisation was not significantly different between the two study groups. This data is noteworthy, as it shows that children with mild COVID-19 and neutropenia do not require longer hospital assistance and strengthens our hypothesis that the occurrence of neutropenia is probably not a negative prognostic factor potentially complicating the course of the disease. Coherently, the clinical course of children included in our study was completely favourable, without the occurrence of any complication or sovrainfection, even in the case of severe neutropenia, and all patients recovered briefly, consistently with other reports [9].

Interestingly, 11 out of 12 of our neutropenic patients were males. A previous study found that neutropenic children are predominantly males and younger, compared with those without this alteration [25]. Regarding COVID-19, a systematic review on haematological alterations in children showed that females had higher leukocyte and neutrophil count [8]. Moreover, a slight prevalence of SARS-CoV-2 infection in male children has been described in previous reports [30]. Considering our small population, this finding is difficult to elucidate; nevertheless, we believe that this remarkable difference represents an interesting aspect that could be investigated in further studies.

Alterations in red blood count (RBC) or haemoglobin level are not common in paediatric COVID-19 [22]. In our study, 3 out of 12 children in each group presented with mild anaemia. This finding is probably more related to the low mean age of our population, as a mild anemization is more frequent in the first months of life [31]. Nevertheless, Henry BM et al. concluded that infants under 1 year of age with mild COVID-19 have significantly reduced levels of haemoglobin [8].

Concerning platelets and clotting profiles, it is known that SARS-CoV-2 infection in adults is associated with major blood hypercoagulability, while in children this represents a rare complication, which occurs mainly in the setting of the related Multisystem Inflammatory Syndrome in Children (MIS-C) [32, 33]. 2 out of 12 (16.6%) of our neutropenic patients presented thrombocytopenia, similarly to a previous retrospective study on paediatric COVID-19 defining rates of 14% [34]. Possible mechanisms of thrombocytopenia in adult COVID-19 include direct infection of the bone marrow, platelet destruction by the immune system, and platelet consumption in course of thrombotic events [35], while in children most of the evidence consists of reports of COVID-19 related immune thrombocytopenia [36, 37]. Platelet count in our patients also recovered spontaneously without further complication.

The favourable clinical course of patients included in our cohort is also coherent with the low levels of inflammatory indices and with the persistently physiological levels of other biochemical indicators in both groups, confirming the absence of multi-organ involvement.

In summary, in our experience, in neutropenic children with mild COVID-19 the absence of complications, the brief duration with spontaneous recovery, the timing of occurrence and resolution confirm the benign and transient nature of this haematological disorder during SARS-CoV-2 infection, as much as in other common viral diseases in children.

A limitation of this study is the small population included, mainly related to the monocentric nature of our investigation; nevertheless, all children admitted to our ward since the beginning of the pandemic were screened and considered for analysis according to inclusion and exclusion criteria. Since we considered only children admitted to our ward, our population is probably under-estimated as it does not include asymptomatic patients or those with milder symptoms who did not require hospitalisation.

## Conclusions

Viral-associated transient neutropenia is a common disorder in paediatric practice and it is usually an incidental finding with a favourable course. Our findings show that the main features of this haematological disorder in COVID-19 are comparable to the well-known transient benign neutropenia associated with other common viral infections. However, we suggest a complete blood count monitoring (1–2 times/week) to confirm the spontaneous recovery of the ANC.

According to our results, it is plausible that neutropenia does not represent a potential negative prognostic factor in paediatric COVID-19. However, these results should be confirmed in further investigation directed towards the following:

- Conducting studies with a more numerous cohort, including children who do not require hospitalisation, to better define the actual prevalence of neutropenia during SARS-CoV-2 infection.
- Longer follow-up of these patients even after discharge, to confirm the benign nature of neutropenia and the absence of long-term complications.

## Data Availability

The datasets used and/or analysed during the current study are available from the corresponding author on reasonable request.

## Abbreviations

SARS-CoV-2: Severe Acute Respiratory Syndrome Coronavirus-2
COVID-19: Coronavirus Disease-19
RT PCR: Reverse-transcriptase polymerase chain reaction
ANC: Absolute Neutrophil Count
WBC: White blood count
CRP: C-reactive Protein
PT: Prothrombin time
APTT: Activated partial thromboplastin time
PEWS: Paediatric Early Warning Score
SD: Standard deviation
CPS1-D: Carbamoyl-phosphate synthetase 1 deficiency
RBC: Red blood count
Hb: Haemoglobin
MIS-C: Multisystem Inflammatory Syndrome in Children
